# Determinants of adverse birth outcome in Tigrai region, North Ethiopia: Hospital-based case-control study

**DOI:** 10.1186/s12887-019-1835-6

**Published:** 2020-01-08

**Authors:** Helen Tsehaye Hailemichael, Gurmesa Tura Debelew, Haileselasie Berhane Alema, Meresa Gebremedhin Weldu, Kebede Haile Misgina

**Affiliations:** 1 Axum Health Center, Tigrai Regional Health Bureau, Tigrai, Aksum, Ethiopia; 2 Department of Population and Family Health, Jimma University, Jimma, Ethiopia; 3 Department of Public Health, College of Health Science, Aksum University, P.O.Box: 298, Axum, Ethiopia

**Keywords:** Adverse birth outcome, Ethiopia, Low birth weight, Preterm birth, Still birth

## Abstract

**Background:**

Adverse birth outcome which attributes to most perinatal deaths is an important indicator of child health and survival. Hence, this study aims to identify determinants of adverse birth outcome among mothers who gave birth in public hospitals of Tigrai region, North Ethiopia.

**Methods:**

Hospital based case-control study was conducted in Tigrai region, Ethiopia between December 2015 and January 2016 among 405 (135 cases and 270 controls) consecutively selected mothers who gave birth in four randomly selected public Hospitals. Mothers with adverse birth outcome (preterm birth; < 37 gestational weeks at birth, low birth weight; < 2.5 kg at birth, or still birth) were the cases while mothers without adverse birth outcome (live birth, birth weight ≥ 2.5 kg and of ≥37 gestational weeks at birth) were the controls. Data were collected by interview and reviewing medical records using structured questionnaire. The collected data were entered into database using EPI info version 3.5.1 then exported to SPSS version 21 for analysis. Finally, multivariate logistic regression was used to identify determinants of adverse birth outcomes at *P* value < 0.05.

**Result:**

The mean age of cases and controls was 27.3 (SD = 6.6) and 26.14 (SD = 4.9) years, respectively. In a multivariate analysis; less than four antenatal care visits [AOR = 4.35, 95% CI: 1.15–13.50], not receiving dietary counseling [AOR = 11.24, 95% CI: 3.92–36.60], not using family planning methods [AOR = 4.06, 95% CI:1.35–17.34], less than 24 months inter pregnancy interval [AOR = 5.21, 95% CI: 1.89–13.86], and less than 11 g/dl hemoglobin level [AOR = 4.86, 95% CI: 1.83–14.01] were significantly associated with adverse birth outcomes.

**Conclusion and recommendation:**

The number of antenatal care visits, ever use of family planning methods, not receiving dietary counseling during antenatal care follow up visits, short inter-pregnancy interval, and low hemoglobin level were identified as independent determinants of adverse birth outcome. A concerted effort should be taken improve family planning use, and antenatal care follow-up with special emphasis to maternal nutrition to prevent adverse birth outcomes.

## Background

Adverse birth outcome defined as low birth weight (LBW); birth weight < 2.5 kg, preterm birth (PTB); < 37 gestational weeks at birth) and stillbirth (fetal death at or after 28 gestational weeks) continues to be public health problem globally mainly in developing countries [1–8]. In 2013, nearly 22 million newborns which accounted for 16% of all babies born globally had low birth weight predominantly in Asia and Africa [8]. Almost all of the stillbirths (98%) occurred in low and middle income countries (LMICs) and nearly three fourth (77%) of the total stillbirths were in Sub-Saharan Africa and South Asia [9, 10]. About half of all stillbirths (1.3 million) occurred during labor and birth [10]. By most recent estimates, 14·9 million babies were born preterm in 2010, accounting for 11.1% of all live births worldwide [2]. According to this study, more than six in ten preterm births were in south Asia and sub-Saharan Africa, where more than half of the global live births occur [2]. Like other developing countries, evidences show that adverse birth outcome is highly prevalent in Ethiopia. For instance, recently conducted study showed that nearly one fourth (23%) of women experience adverse birth outcomes (14.3% preterm, 11.2% low birth weight and 7.1% stillbirth) [11].

Though adverse birth outcomes and their consequences are preventable [1, 6, 12, 13], preterm birth and low birth weight are major determinants of perinatal and child mortality and for survivors long term adverse health consequences mainly in low and middle income countries (LMICs) [1, 4, 5, 7, 14–19]. That is, preterm birth has significant contribution in child mortality. Complications of preterm birth caused 15.4% of the estimated 6.3 million under five children who died in 2013. Preterm birth is the leading cause of death amongst neonates (death in the first 28 days of life) [1]. Similarly, children with low birth weight have higher risk of perinatal death (still birth and neonatal death), and post-neonatal death [5, 7, 19, 20]. For survivors, preterm birth and low birth weight increases risk of stunting (chronic malnutrition), metabolic disorders and chronic non-communicable diseases [16, 18].

Different studies revealed that different factors could contribute for adverse birth outcome; for instance socio-demographic characteristics such as residence [21], age, marital status, education [22], occupation [23], and low socioeconomic status [24]. Related to reproductive and obstetric characteristics; parity, gravidity, birth interval [25], pregnancy plan [26], maternal nutritional status [2, 21, 23, 24, 27], past history of stillbirth, age of sexual debut [23], prolonged and obstructed labor [28], caesarean section delivery [19], history of preterm birth antepartum hemorrhage, and history of perinatal death [11]. Besides to this, maternal health service related factors like longer distance to walk to health facilities [21, 24], and lack of antenatal care follow-up [11, 20, 22, 24]. Additionally environmental factors such as water supply [29], washing hands with water only, not having separate kitchen room, using firewood for cooking, and using kerosene for cooking [30] could predict adverse birth outcomes.

To investigate the problem, epidemiological data pertaining to determinants of adverse birth outcome are required for planning and implementing proper interventions. However, determinants of adverse birth outcomes are poorly documented and understood in the study area. Therefore, local epidemiological studies are fundamental, to assess determinants of adverse birth outcome and identify target areas for future interventions. Thus, this hospital based case control study aims to identify determinants of adverse birth outcome among mothers who gave birth in public hospitals, Tigrai region, North Ethiopia.

## Methods

### Study design, setting and population

Hospital based unmatched case-control study was conducted from December 2015 to January 2016 among mothers who gave birth in four randomly selected public hospitals to identify determinants of adverse birth outcome. The study was conducted in Tigrai region, northern Ethiopia. The region is administratively divided into 7 zones, 52 woredas and 814 Kebeles (kebele is the smallest administrative unit in Ethiopia). In Tigrai regional state, there are 2 comprehensive specialized hospitals, 15 general hospitals, 20 primary hospitals, 204 health centers, 712 health posts and over 500 private health facilities. Our study was conducted in general hospitals. General hospitals provide inpatient and outpatient services, including maternal and child care. General hospitals also serve as a referral center for primary hospitals. Hence the setups of the general hospitals are similar in kind of services they deliver, health information recording system, and in number and qualification of health professional required.

According to the 2007 census projection, the total population of Tigrai regional state is 5,055,999 (50.8% females and 49.2% males). A total estimated number of 116,506 births have been registered annually in the region. For this particular study, the source population was all mothers who gave birth and infants in pair in general hospitals in Tigrai region, Northern Ethiopia. Cases were mothers with adverse birth outcome (preterm birth; < 37 gestational weeks at birth, low birth weight; weight of < 2.5 kg at birth, or stillbirth), and controls were mothers without adverse birth outcome (live birth, weight ≥ 2.5 kg at birth and ≥ 37 gestational weeks at birth).

### Sample size and sampling procedure

The sample size was determined using EPI-Info 3.5.1 statistical software. The assumptions for the sample size calculation were: proportion of non-educated mothers among controls [30], minimum detectable odds ratio of 2, confidence level of 95%, power of 80%, a case to control ratio of 1:2 and non-response rate of 5%. The total sample size was 418 (139 cases and 279 controls). From the public hospitals that provide institutional delivery services in Tigrai region, four hospitals were selected randomly. Considering the number of mothers who gave birth at each of the selected hospitals in four months prior to the data collection time, the average number of mothers expected to give birth per month in each of the respective hospitals was estimated. Then, the total sample size was allocated proportionally to each of the four randomly selected hospitals. Finally, cases and controls were selected consecutively until the allocated sample was reached at each hospital. Eventually, a total of 405 mothers (135 cases and 270 controls) were participated in this study.

### Data collection tool and procedure

Data were collected by interview using structured questionnaire adopted from Ethiopian Demographic and Health Survey and other related literatures [11, 20–22, 25, 26, 28, 30–32]. In addition, medical records were also reviewed. The questionnaire was translated into Tigrigna, which is the local language of the study area. The contents of the questionnaire included: socio-demographic and economic characteristics, reproductive and obstetric factors, maternal service utilization and birth outcomes. Prior to data collection, the questionnaire was pre-tested on 20 of mothers who gave birth at Axum St. Marry Hospital. Necessary modifications were made based on the nature of gap identified in the questionnaire. Mothers with their newborn pairs were the study participants. The mothers were interviewed and birth weight of the newborn was evaluated by trained midwives. The socio-demographic characteristics, reproductive and obstetric factors, and maternal health services information was collected from the mothers whereas birth weight was measured from newborns. Furthermore, the data collection process was closely monitored by the principal investigators. Both data collectors and supervisors were trained for two days on the objectives of the study, sampling technique, ethical consideration, data collection tool and techniques of collecting data to maintain precaution throughout the study. The collected data were checked daily for completeness by the supervisors and feedback was provided to data collectors when necessary.

The weight of the newborns was measured using a Salter’s hang-up scale to the nearest 100 g following standard techniques within 15 min after delivery and basic newborn care has been given. The newborns were weighted naked or in minimal clothing. Maternal MUAC (mid upper arm circumference) was also measured following the appropriate procedure to the nearest centimeter using the standard measuring tape.

### Data processing and analysis

Data were coded, cleaned, edited and entered to EPI info version 3.5.1 and exported to SPSS version 21 for analysis. Descriptive statistics such as means and proportions were used to summarize the data. In this study, cases were coded as 1 and controls were coded as 0. Bivariate logistic regression analysis was used to see the unadjusted effect of each independent variable on the dependent variable of the study. Considering all independent variables with a *p* < 0.25 in the bivariate analyses as candidates, multivariable logistic regression model was fitted to identify independent determinants of adverse birth outcome. Finally, variables at a *P* < 0.05 were considered statistically significant in the final multivariable logistic regression model. Model fitness was checked by Hosmer and Lemeshow (*P* value = 0.39). Adjusted odds ratio (AOR) with 95% CI was computed to determine the strength of association between the variables of interest.

## Results

### Socio - demographic characteristics of participants

A total of 405 mothers (135 cases and 270 controls) were included in the present study. The cases were consists of low birth weight, preterm and stillbirth; 78 (57.8%), 45 (33.3%) and 12 (8.9%) respectively. The mean age of cases and controls with standard deviation (SD) was 27.3 ± 6.6 and 26.14 ± 4.9 years, respectively. Majority of mothers, 84.4% of the cases and 93.0% of the controls were married. Among the total mothers included in the study, 46 (34.1%) of the cases and 37 (13.7%) of the controls were unable to read and write. Most mothers, 72.6% of the cases and 70% of the controls were housewives. Likewise, majority of the cases (89.6%) and the controls (88.9%) were Orthodox Christian by religion. Seventy seven (57%) of the cases and 221 (81.5%) of the controls were living in urban (Table 1).

**Table 1:** Socio-demographic characteristics of mothers who gave birth in public hospitals, Tigrai region, Ethiopia, December 2015–January 2016 (*n* = 405; cases: 135 and controls: 270)

Variables	Categories	Cases *n* = 135 n (%)	Controls *n* = 270 n (%)
Maternal age (years)	15–19	10 (7.4)	12 (4.5)
	20–24	48 (35.6)	91 (33.7)
	25–29	30 (22.2)	107 (39.6)
	30–34	19 (14.1)	40 (14.8)
	≥35	28 (20.7)	20 (7.4)
Marital status	Single	14 (10.4.0)	11 (4.0)
	Married	114 (84.4)	251 (93.0)
	Others	7 (5.2)	8 (3.0)
Maternal education	Unable to read and write	49 (36.3)	34 (12.6)
	Able to read and write only	12 (8.9)	26 (9.5)
	Primary school	27 (20)	77 (28.5)
	Secondary school	30 (22.2)	77 (28.5)
	College and above	17 (12.6)	56 (20.7)
Husband’s education	Unable to read and write	37 (27.4)	21 (7.8)
	Able to read and write only	21 (15.5)	29 (10.7)
	Primary school	22 (16.3)	53 (19.6)
	Secondary school	33 (24.5)	84 (31.1)
	College and above	22 (16.3)	83 (30.8)
Occupation	House wife	98 (72.6)	189 (70.0)
	Student	10 (7.4)	9 (3.3)
	Employed	27 (20.0)	72 (26.7)
Religion	Orthodox	121 (89.6)	249 (92.2)
	Muslim	12 (8.9)	14 (5.2)
	Catholic	2 (1.5)	7 (2.6)
Residence	Urban	77 (57)	221 (81.9)
	Rural	58 (43)	49 (18.1)
Monthly household income (ETB) *	≤650	37 (27.4)	14 (5.2)
	651–1400	35 (25.9)	53 (19.6)
	1401–2350	24 (17.8)	63 (23.3)
	2351–3550	26 (19.2)	71 (26.3)
	3551–5000	12 (8.9)	49 (18.2)
	≥5001	1 (0.8)	20 (7.4)

*1ETB ~ O.045 USD

Obstetric characteristics of participants

Most of the participants, 66% cases and 80% controls had a total lifetime gravidity of less than four. Ninety three (68.9%) of cases and 221 (81.8%) of controls had less than four parity. Among participants of the study, 61 (70.1%) cases and 40 (27.6%) controls had less than 24 months inter-pregnancy interval. Majority of the participants of the study, 110 (81.5%) cases and 270 (100%) controls attended ANC at least once in health facility. Meanwhile, mothers who had at least four ANC visits were 40 (29.6%) among cases and 195 (72.2%) among controls. Among the total participants 88 (65.2%) of the cases and 30 (11.1%) of the controls did not receive dietary counseling during the index pregnancy. Among the total mothers included in the present study, 76 (56.3%) of the cases and 235 (87%) of the controls had hemoglobin level of 11 g/dl or more during the index pregnancy. In addition, in 50 (37%) of the cases and 28 (10.4%) of the controls the index pregnancy was unwanted (Table 2).

**Table 2:** Obstetric characteristics of mothers who gave birth in public hospitals, Tigrai region, Ethiopia, 2016

Variables	Categories	Cases (135) n (%)	Control (270) n (%)
Gravidity	< 4	89 (66)	216 (80)
	≥ 4	46 (44)	54 (20)
Parity	< 4	93 (68.9)	221 (81.8)
	≥ 4	42 (31.1)	49 (18.2)
Antenatal care (ANC) follow up	No	25 (18.5)	0 (0.0)
	Yes	110 (81.5)	270 (100)
Gestational age (GA) at first ANC visit	< 28 weeks	75 (68.2)	249 (92.2)
	≥28 weeks	35 (31.8)	21 (7.8)
Number of ANC visits	< 4	95 (70.4)	75 (27.8)
	≥ 4	40 (29.6)	195 (72.2)
Iron folic acid (IFA) supplementation	No	46 (34.1)	31 (11.5)
	Yes	89 (65.9)	239 (88.5)
Amount of IFA taken	< 90	59 (80.8)	133 (61.9)
	90 and more	14 (19.2)	82 (38.1)
Received dietary counseling	No	88 (65.2)	30 (11.1)
	Yes	47 (34.8)	240 (88.9)
Hemoglobin level	< 11 g/dl	59 (43.7)	35 (13.0)
	≥ 11 g/dl	76 (56.3)	235 (87.0)
MUAC	< 21 cm	83 (61.5)	3 (1.1)
	≥ 21 cm	52 (38.5)	267 (98.9)
Maternal height	< 150 cm	15 (11.1)	11 (4.1)
	≥ 150 cm	120 (88.9)	259 (95.9)
Maternal RH factor	Positive	123 (91.1)	252 (93.3)
	Negative	12 (8.9)	18 (6.7)
Pregnancy wanted/planned	No	50 (37.0)	28 (10.4)
	Yes	85 (63.0)	242 (89.6)
Ever use of family planning methods	No	75 (55.5)	61 (22.6)
	Yes	60 (44.5)	209 (77.4)
History of medical illness	No	123 (91.1)	253 (93.7)
	Yes	12 (8.9)	17 (6.3)
Current labor complication	No	110 (81.5)	219 (81.1)
	Yes	25 (18.5)	51 (18.9)
Sex of index neonate	Male	69 (51.1)	132 (48.9)
	Female	66 (48.9)	138 (51.1)
APGAR score	< 8	84 (62.2)	57 (21.1)
	8 and more	51 (37.8)	213 (78.9)

Determinants of adverse birth outcome

In the bivariate analysis, factors found to be significantly associated with adverse birth outcome were maternal education, residence, husband’s education, monthly household income, gravidity, inter-pregnancy birth interval, number of ANC visits, iron folic acid supplementation (IFA), receiving dietary counseling during the prenatal care of the index pregnancy, ever use of family planning, wanted/plan of the index pregnancy, experience complication in the index pregnancy, and hemoglobin level.

After controlling for confounders using multivariate analysis, number of ANC visit, receiving dietary counseling or not during the index pregnancy, inter-pregnancy interval, ever use of family planning methods, and hemoglobin level were identified as independent determinant factors of adverse birth outcome. That is; mothers who had less than four ANC visits were about four times more at risk of experiencing adverse birth outcome than mothers who had at least four ANC visit [AOR = 4.35, 95% CI:1.15–13.50]. Mothers who did not receive dietary counseling were 11 times more at risk of experiencing adverse birth outcomes compared to their counterparts, [AOR = 11.24, 95% CI: 3.92–36.60]. Furthermore, mothers with hemoglobin level of less than 11 g/dl were nearly five times more at risk to experience adverse birth outcome than those with hemoglobin level of 11 g/dl or more [AOR = 4.86, 95% CI: 1.83–14.01]. Ever use of family planning methods was also another important variable independently associated with adverse birth outcome; mothers who had not ever used family planning methods were four times more likely to experience adverse birth outcome compared to their counterparts, [AOR = 4.06, 95% CI: 1.35–17.34]. Mothers with less than 24 months inter pregnancy interval were five times more risk to experience adverse birth outcome than mothers with greater than 24 months inter-pregnancy interval, [AOR = 5.21, 95% CI: 1.89–13.86 (Table 3).

**Table 3:** Determinants of adverse birth outcome among mothers who gave birth in public hospitals, Tigrai region, Ethiopia, 2016 (n = 405)

Variables	Cases	Controls	COR (95% CI)	AOR (95% CI)*
	n (%)	n (%)		
Maternal education				
Non formal educated	61 (45.2)	60 (22.2)	2.86 (3.01–8.59)	0.62 (0.16–2.41)
Formal educated	74 (54.8)	210 (77.8)	1	1
Residence				
Urban	77 (25.8)	221 (74.2)	0.29 (0.19–0.47)	0.50 (0.12–2.09)
Rural	58 (54.2)	49 (45.8)	1	1
Monthly household income				
< 1200	72 (51.8)	67 (48.2)	5.66 (2.97–10.78)	0.97 (0.20–4.76)
1200–3000	48 (27.9)	124 (72.1)	2.04 (1.07–3.88)	1.05 (0.29–3.71)
> 3000	15 (16.0)	79 (84.0)	1	1
Husband’s education				
Non formal educated	58 (53.7)	50 (46.3)	3.31 (2.09–5.24)	1.08 (0.28–4.25)
Formal educated	77 (25.9)	220 (74.1)	1	1
Gravidity				
1–3	89 (29.2)	216 (70.8)	0.48 (0.30–0.77)	1.78 (0.67–4.76)
≥ 4	46 (46.0)	54 (54.0)	1	1
Number of ANC visits				
< 4	95 (55.9)	75 (44.1)	6.17 (3.92–9.74)	4.35 (1.15–13.50)*
≥ 4	40 (17.0)	195 (83.0)	1	1
Received dietary counseling				
No	88 (74.6)	30 (25.4)	14.98 (8.91–25.17)	11.24 (3.92–36.60)***
Yes	47 (16.4)	240 (83.6)	1	1
Ever use of family planning methods				
No	75 (55.1)	61 (44.9)	4.28 (2.75–6.67)	4.06 (1.35–17.34)*
Yes	60 (22.3)	209 (77.7)	1	1
Wanted /planed the indexed pregnancy				
No	50 (64.1)	28 (35.9)	5.08 (3.01–8.59)	1.46 (0.40–5.33)
Yes	85 (26.0)	242 (74.0)	1	1
Experienced pregnancy complication				
No	103 (29.7)	244 (70.3)	0.34 (.19–.60)	0.61 (0.15–2.43)
Yes	32 (55.2)	26 (44.8)	1	1
Hemoglobin level				
< 11 mg/dl	59 (62.8)	35 (37.2)	5.21 (3.19–8.52)	4.86 (1.83–14.01)**
≥ 11 mg/dl	76 (24.4)	235 (75.6)	1	1
Received IFA				
No	46 (59.7)	31 (40.3)	3.98 (2.38–6.68)	0.67 (0.18–2.57)
Yes	89 (27.1)	239 (72.9)	1	1
Inter- pregnancy interval				
< 24 months	61 (60.4)	40 (39.6)	6.16 (3.43–16.06)	5.21 (1.89–13.86)***
> = 24 months	26 (19.8)	105 (80.2)	1	1

*** Significant at P- value < 0.001, **significant at P- value < 0.01 and *significant at P- value < 0.05

*Adjusted for: number of ANC visits, iron folic acid (IFA) supplementation, dietary counseling, ever use family planning, inter-pregnancy interval, and hemoglobin level

## Discussion

This study assessed determinants of adverse birth outcome among mothers who gave birth in public hospitals in Tigrai region, North Ethiopia. the results revealed that limited number of ANC visit, not receiving dietary counseling, short inter-pregnancy interval, not ever using of family planning methods, and low hemoglobin level were independently associated with adverse birth outcome.

In the present study, mothers who had less than four ANC follow-up visits had four times more risk of having adverse birth outcome than those who have had at least four ANC follow up visits. This finding is similar with a similar study in Gaza strip [31], in which the risk of preterm birth increased two folds among mothers who had less than four ANC visits compared to their counterparts. Similarly, the current finding is supported by similar studies in Ethiopia and India [24, 26, 30, 33–35], in which not having ANC has been shown as a significant risk factor of low birth weight. Furthermore, the current finding is in agreement with similar studies in Ethiopia and China, and a systematic review in LIMICs [11, 17, 20, 22, 36], in which the risk of still birth was significantly higher among mothers who had no ANC visits compared to their counterparts. This could be explained by mothers who have the recommended number of ANC visits have access to information on maternal nutrition which eventually results in better dietary practice. This finding implies that adverse birth outcomes could be reduced by improving maternal service utilization.

Dietary counseling was another independent predictor of adverse birth outcome; mothers who did not receive dietary counseling during the index pregnancy had twelve fold increased risk of experiencing adverse birth outcome than those who received dietary counseling. This finding is supported by a recently conducted systematic review [37]. This suggests that antenatal care should give much more emphasis to dietary counseling with special focus to stunted mothers who are at increased risk of adverse birth outcome. Besides to dietary counseling, hemoglobin level was associated with adverse birth outcome; mothers with hemoglobin level of less than 11 g/dl had five times increased risk of adverse birth outcome compared to their counterparts. This is supported by similar studies in India, Nigeria, Sudan and Ethiopia [19, 36, 38–42], in which low hemoglobin level has been reported as a risk factor of low birth weight and/or preterm birth and/or still birth. This could be explained by the fact that hemoglobin level is a proxy indicator of nutritional status which significantly influence birth outcome due to low fetal supply of oxygen and nutrient. This implies that maternal interventions should primarily focus on maternal nutrition and iron folic acid supplementation.

Mothers with short (less than 24 months) inter-pregnancy interval were five times more likely to experience adverse birth outcome compared to their counterparts. This result is in line with studies in Indonesia, India, Tanzania and Ethiopia [25, 41, 43–46], in which short inter-pregnancy interval was a risk factor for low birth weight and/or preterm birth. Likewise, the current finding is in agreement with similar study in Bangladesh [47], in which short inter- pregnancy interval has been a significant risk factor for stillbirth. This could be due to the fact that short inter-pregnancy interval results in maternal depletion syndrome which eventually increases the risk of adverse birth outcome [48]. Furthermore, ever use of family planning was significantly associated with adverse birth outcome; mothers who have not ever used family planning methods had five times more risk to experience adverse birth outcome than their counterparts. This is supported by a recently conducted systematic review and meta-analysis study [46], in which continuum of care – linking pre-pregnancy care and pregnancy care has been found to be effective in reducing adverse birth outcome. This could be explained by the fact that not using family planning may results in too early, too many and too close births which increases the risk of maternal depletion and adverse birth outcome [49, 50]. The implication of this finding is interventions targeted adverse birth outcomes should give emphasis to family planning [51].

This study has its own drawbacks. The study did not assess the contribution of important variables such as knowledge, husband involvement and food security. Furthermore, this study is not free from recall and social desirability biases.

## Conclusion

In this study less than four ANC visits, not receiving dietary counseling during the index pregnancy, not ever using family planning methods, inter-pregnancy interval of less than 24 months, and hemoglobin level of less than 11 g/dl were significantly associated with adverse birth outcomes. Therefore, a concerted effort should be taken to improve family planning, and ANC follow-up with special emphasis to maternal nutrition to prevent adverse birth outcome.

## Data Availability

Datasets obtained or analyzed during the current study are available from the corresponding author on reasonable request.

## Abbreviations

ANC: Antenatal care
AOR: Adjusted odds ratio
CI: Confidence interval
COR: Crude odds ratio
ETB: Ethiopian birr
GA: Gestational age
IFA: Iron Folic Acid
LBW: Low birth weight
LMICs: Low and middle income countries
MUAC: Mid upper arm circumference
PTB: Preterm birth
SD: Standard deviation
WHO: World Health Organization
